# Additional intraoral radiographs may change the judgment regarding the final position of orthodontic mini-implants

**DOI:** 10.1590/2177-6709.23.2.054-061.oar

**Published:** 2018

**Authors:** Marina K. Oba, Guido A. Marañón-Vásquez, Fábio L. Romano, Christiano Oliveira-Santos

**Affiliations:** 1 Universidade de São Paulo, Hospital das Clínicas, Faculdade de Medicina de Ribeirão Preto, Residência em Atenção Multiprofissional ao Câncer (Ribeirão Preto/SP, Brazil).; 2 Universidade de São Paulo, Faculdade de Odontologia de Ribeirão Preto, Departamento de Clínica Infantil, Ortodontia (Ribeirão Preto/SP, Brazil).; 3 Universidade de São Paulo, Faculdade de Odontologia de Ribeirão Preto, Departamento de Estomatologia, Saúde Coletiva e Odontologia Legal, Radiologia (Ribeirão Preto/SP, Brazil).

**Keywords:** Dental radiography, Bitewing radiography, Bone screws.

## Abstract

**Objective:**

This study aimed to assess if additional vertical bitewing (VBW) and/or occlusal (OC) radiographs may change initial judgment based only on periapical radiograph (PAR) about the final position of orthodontic mini-implants (OMI).

**Methods:**

Subjective and objective analyses were performed. Radiographic images of 26 OMI were divided into four groups: PAR, PAR+VBW, PAR+OC and ALL (PAR+VBW+OC). For subjective analysis, five observers were asked to assess if the position of OMI was favorable to its success, using questionnaires with a four-point scale for responses: 1= definitely not favorable, 2= probably not favorable, 3= probably favorable, or 4= definitely favorable. Each group containing sets of images was presented to them in four different viewing sessions. Objective evaluation compared horizontal distances between OMI tip and the root nearest to the device in PAR and VBW.

**Results:**

Most of observers (3 out of 5) changed their initial judgment based on PAR about OMI position when additional radiographs were analyzed. Differences between groups (i.e. PAR *vs.* PAR+VBW; PAR *vs.* PAR+OC; and, PAR*vs.*ALL) were statistically significant for these observers. For those that changed their judgment about OMI position, confidence level could significantly increase, decrease or even be maintained, not indicating a pattern. There was no agreement for distances between OMI tip and the root nearest to the device in PAR and VBW.

**Conclusion:**

Considering the limitations of the study, it is concluded that additional radiographic images may change the judgement about OMI final position without necessarily increasing the degree of certainty of such judgment.

## RESUMO

**Objetivo:**

avaliar se a adição de radiografias interproximais verticais (IV) e/ou oclusais (OC) pode alterar o julgamento inicial sobre a posição final dos mini-implantes ortodônticos (MI) baseado somente na radiografia periapical (PA).

**Métodos:**

foram realizadas análises subjetivas e objetivas. Imagens radiográficas de 26 regiões contendo MI foram divididas em quatro grupos: PA, PA+IV, PA+OC e TODAS (PA+IV+OC). Na análise subjetiva, cinco observadores foram convidados a avaliar se a posição do MI era favorável para o seu sucesso, utilizando questionários com uma escala de quatro pontos para respostas: 1 = definitivamente não favorável; 2 = provavelmente não favorável; 3 = provavelmente favorável; ou 4 = definitivamente favorável. Cada grupo contendo conjuntos de imagens foi apresentado aos observadores em quatro sessões diferentes. Adicionalmente, uma avaliação objetiva comparou as distâncias horizontais entre a ponta do MI e a raiz dentária mais próxima ao dispositivo na PA e IV.

**Resultados:**

a maioria dos observadores (3 de 5) mudou seu julgamento inicial sobre a posição do MI baseado na PA quando radiografias adicionais foram analisadas. Diferenças entre os grupos (ou seja, PA *vs* PA+IV; PA *vs* PA+OC; e PA*vs*TODAS) foram estatisticamente significativas para esses observadores. Para aqueles que mudaram seu julgamento sobre a posição do MI, o nível de confiança das respostas aumentou, diminuiu ou foi mantido, não indicando um padrão. Houve diferença estatisticamente significante entre as distâncias da ponta do MI para a raiz mais próxima ao dispositivo na PA e IV.

**Conclusão:**

considerando-se as limitações desse estudo, concluiu-se que imagens radiográficas adicionais podem alterar o julgamento sobre a posição final de MI sem, necessariamente, aumentar o grau de certeza de tal julgamento.

## INTRODUCTION

Correct positioning of orthodontic mini-implants (OMI) is one of the most important factors associated to their success rate.^1^ ^-^ ^5^ Several methods^6^ ^-^ ^17^ have been used to assess the position of these devices. Periapical radiograph (PAR) is the most used imaging modality for post-placement evaluation of OMI position,^18^ despite its limitations and the lack of studies to actually support this recommendation. 

Three-dimensional imaging, such as cone beam computed tomography (CBCT), could allow a more accurate observation of the relationship between the OMI and the roots.^16^ In fact, significant differences between PAR and CBCT have been found regarding the assessment of the proximity of the OMI and roots, with less than 50% agreement between these imaging modalities.^17^ ^,^ ^19^ However, although CBCT seems to be the most indicated method for this assessment,^4^ ^,^ ^5^ ^,^ ^20^ higher radiation dose and cost are still disadvantages that preclude their routine use. This justifies the search for other radiographic methods with the potential to assist or complement the assessment of OMI final position when necessary. Vertical bitewing technique (VBW) has been used as preoperative radiography to ensure precise mapping of the sites for OMI insertion and also as alternative evaluation method after its placement,^21^ ^-^ ^23^ presenting less distortion than PAR^15^ and preventing root projection on interradicular bone, avoiding thus incorrect or limited image interpretation.^24^ On the other hand, occlusal radiography (OC) shows a totally different perspective of the device and related structures around it.^14^

Additional radiographic images may assist clinicians, however, those could also mislead them, changing their opinions without necessarily increasing the confidence level of their judgments. The present study aimed to assess if additional radiographs (VBW, OC or both) may change the initial judgment based on PAR only, about the position of OMI. The null hypothesis tested was that there is no difference between OMI position evaluation using only PAR or adding other radiographs. Objective comparative analysis between distance measurements on PAR and VBW was also performed to support or not the results of subjective evaluation.

## MATERIAL AND METHODS

Institutional Research Ethics Committee approved the study (protocol #56317015.5.0000.5419). Patients in treatment at the Orthodontics Graduate Clinic who had indication of buccal OMI placement between two adjacent teeth in the posterior upper or lower regions were selected. Participants had to have permanent dentition and should not have fixed appliances in palatal or lingual regions. Informed consent was obtained from patients before clinical procedures.

A sample size calculation was performed based on the reported results of a previous published article.^15^ A α error probability of 0.05 and power of 0.8 were used for an estimate of difference between PAR and VBW in 45% of the cases, resulting in a sample size of at least 20 mini-implants. G*Power 3.1.9 software (http://www.gpower.hhu.de/en.html) was used for calculation.

**Figure 1:**
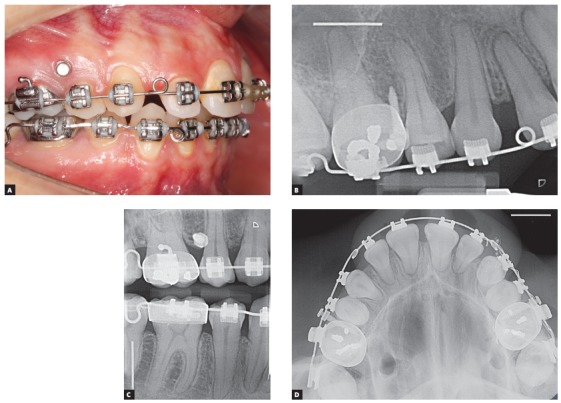
A) Orthodontic mini-implant (OMI) placed between upper second premolar and first molar through the buccal attached gingiva: B) periapical radiograph (PAR); C) vertical bitewing radiograph (VBW); and D) occlusal radiograph (OC).

Twenty-six OMI (Conexão, Arujá, São Paulo, Brazil) (14 maxillary, 12 mandibular) were included for radiographic analysis. Self-tapping devices (diameter, 1.5 mm; length, 8 mm; transmucosal profile, 1 mm) were implanted in the posterior regions through the buccal attached gingiva into the interradicular space of the indicated teeth in the maxillary and mandibular arches of eight patients (Fig 1A). The same operator installed all devices. 

After implantation, digital radiographs were performed using Vistascan phosphor-plate digital system (Dürr Dental AG, Bietigheim-Bissingen, Germany). PAR (by paralleling technique) and VBW were performed using XCP-ORA and XCP-DS sensor positioning system (Dentsply RINN, York, PA, USA) convenient for each type of radiograph to standardize both techniques. For maxillary OC, the patient was positioned with the Camper’s plane parallel to the floor and the central beam oriented to the nasal dorsum at a 65^o^ angle. For OC of the mandible, the central beam was directed to the center of the floor of the mouth with a perpendicular orientation to the sensor. Exposure time ranged from 0.16 to 0.25 seconds. Phosphor plates were exposed at 55 kV and 10 mA. Images were then exported through the software DBSWIN 5.3.1 (BDSWIN, Dürr Dental, AG, Bietigheim-Bissingen, Germany) in tiff format for further evaluation. Each type of technique (PAR, VBW and OC) was performed for all OMI (Figs 1B, 1C and 1D) by the same operator. Subjective and objective radiographic evaluations were subsequently realized.

### Subjective radiographic evaluation

Five orthodontists performed subjective analysis. Radiographs of the 26 OMI were divided into four groups: PAR (PAR only), PAR+VBW, PAR+OC, and ALL (PAR, VBW, and OC). The sets of radiographs were presented to the observers in four different viewing sessions with an interval of at least 15 days between them. PowerPoint presentations containing the sequence of radiographic images selected for analysis in personal computer were sent for each observer via Dropbox (Dropbox Inc, San Francisco, California, USA). Brightness, contrast and size of each image could be modified by each one of them according to their suitability for a correct analysis. Observers also received, via email, a questionnaire with closed questions to be resolved in an approximate time of 10 to 15 minutes (once the analysis started) without the intervention of other observer. Subsequently, the questionnaire was returned by the same route. At this moment, it began the interval of 15 days for a new analysis.

Observers were asked to assess whether the position of the OMI was favorable to its success, based on the analysis of the images only. A four-point scale was created for this purpose: 1= Definitely not favorable, 2= Probably not favorable, 3= Probably favorable, 4= Definitely favorable. On the first viewing session, PAR of each OMI was presented for them. In second and third sessions, radiographs from groups PAR+VBW and PAR+OC were alternately presented. In the fourth viewing session all radiographs from each OMI were presented (ALL group). The order of presentation of the radiographs in every session was random (sequence generated at http://random.org.). 

### Objective radiographic evaluation

**Figure 2:**
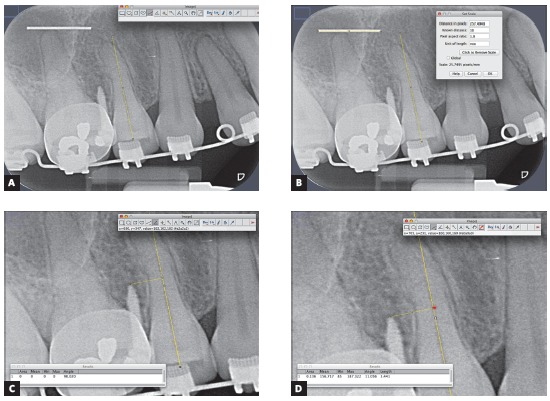
Objective evaluation in PAR using Image J software: A) long axis of the reference dental root (yellow line); B) measurement parameters calibration; C) 90° angle formed by root long axis line and the measurement line; D) intersection point of two lines (red point) and distance between OMI tip and root long axis.

Objective evaluation compared only PAR and VBW. The analyses were performed using Image J software (NIH, Bethesda, MD, USA) by two trained and calibrated observers who performed measurements of the distances between the OMI tip and the long axis of the root nearest to the device. The same reference dental root was considered for measurements on both radiographs (nearest root was defined in PAR). The long axis of the selected root was represented by a line that passed through the middle of the root canal in its cervical and middle thirds, marked using the “straight tool” on the tool bar of the software (Fig 2A). This image (with the root long axis designed) was saved for further analysis.

Measurement parameters were adjusted using a 10-mm orthodontic wire previously placed on the radiographic sensor. Distance in pixels provided by the software was converted into millimeters based on known length of the wire (Fig 2B).

To measure the distance between the OMI tip and the root, a line was drawn at an angle of 90^o^ (± 0.5^o^) with the previously defined root long axis using the “angle” and “paintbrush” tools (Fig 2C). Then the distance between the OMI tip and the long axis line was measured and registered in millimeters (measurements were made up to the second decimal place) (Fig 2D). Brightness, contrast and size of each image could be modified according to the observer’s suitability for a correct analysis.

For calibration purposes, any measurement with 10% or higher difference between observers was revised.

### Statistical analysis

For subjective analysis, interobserver agreement was assessed by weighted Kappa index (Kw), which was interpreted as low (0 - 0.2), reasonable (0.21 - 0.4), moderate (0.41 - 0.6), substantial (0.61 - 0.8) and very high agreement (0, 81 - 1.0). The responses of observers were compared for each group. 

Subjective analysis consisted of two different perspectives: judgment of the OMI position (not favorable or favorable, i.e. 1 + 2 and 3 + 4 in the four-point scale, respectively) and the confidence level of responses (more confident or less confident, i.e. 1 + 4 and 2 + 3 in the four-point scale, respectively). McNemar test was used to evaluate if there were differences between the groups.

Intraclass correlation coefficient (ICC) was calculated to determine interobserver agreement in the objective analysis. Later, using the mean values of two observers for each measurement, ICC was again used to assess the agreement between measurements obtained with these different instruments (PAR and VBW). In addition, the frequency of cases with substantial difference (i.e. greater than 0.5 mm) between PAR and VBW regarding the distance between OMI tip and the nearest root was calculated.

Data were analyzed using PASW Statistics software (version 17.0; SPSS Inc., Chicago, IL, USA). Kw was calculated online with GraphPad QuickCalcs (www.graphpad.com/quickcalcs). The level of statistical significance adopted was 5%.

## RESULTS

### Subjective evaluation

Interobserver agreement (Kw) for groups PAR and PAR+VBW ranged from low to substantial (0.02 - 0.66 and 0.01 - 0.6, respectively); for group PAR+OC, from low to moderate (0.03 - 0.55); and, for group ALL, from low to reasonable (0.08 - 0.39). There was a statistically significant difference between groups PAR and PAR+OC (mean 0.27 and 0.16, respectively).

Frequencies of responses for each observer regarding the OMI position and confidence level of responses are shown in Table 1. Most of observers (3 out of 5) changed their initial judgment based on PAR only about OMI position when additional radiographs were analyzed (i.e. PAR *vs.* PAR+VBW; PAR *vs.*PAR+OC; or, PAR *vs.* ALL). 

**Table 1:** Subjective analysis. Frequency (%) of observer responses regarding the judgment of favorable OMI position and their confidence levels.

Subjective Analysis	Observer	Groups			
		PAR	PAR + VBW	PAR + OC	ALL
Favorable OMI position	1	81.0%^b^	76.9%^b^	92.3%^a^	76.9%^b^
	2	73.1%^a^	65.4%^a^	80.8%^a^	80.8%^a^
	3	96.2%^a^	76.9%^b^	92.3%^a^	65.4%^b^
	4	19.2%^b^	26.9%^a^	30.8%^a^	23.1%^a^
	5	46.2%^b^	69.2%^a^	76.9%^a^	80.8%^a^
High confidence level of responses	1	53.9%^a^	42.3%^a^	30.8%^b^	42.3%^a^
	2	15.4%^b^	34.6%^a^	19.2%^b^	57.7%^a^
	3	53.9%^a^	65.4%^a^	46.2%^a^	53.9%^a^
	4	69.2%^a^	42.3%^b^	42.3%^b^	46.2%^b^
	5	42.3%^b^	76.9%^a^	61.5%^a^	65.4%^a^

Different letters (a and b) represent statistically significant difference (McNemar test; p<0.05) between groups for each observer.

When the VBW was added (PAR *vs.* PAR + VBW), observers 3, 4 and 5 changed their opinion; when OC was added (PAR *vs.* PAR + OC), observers 1, 4 and 5 modified their judgment; and, when both radiographs were added (PAR *vs.* ALL), observers 3, 4 and 5 modified their opinion on whether or not the position was favorable to the success of the device. Differences between groups were statistically significant for these observers. 

Regarding the analysis of confidence level of responses, the results were highly variable. For the observers that changed their judgment about OMI position, confidence level could significantly increase, decrease or even be maintained without indicate a pattern. 

### Objective evaluation

Interobserver agreement was high for PAR (ICC= 0.98) and VBW (ICC= 0.99). Differences between PAR and VBW in the distances measured from OMI tip and the nearest root ranged from 0.05 to 2.54 mm (mean difference 1.05 mm). Degree of agreement between measurements obtained with both instruments was low (ICC= 0.22).

In 7 cases, PAR and VBW yielded similar distance measurements. However, distances were greater in PAR or VBW in 9 and 10 cases, respectively (Table 2).

**Table 2:** Objective analysis. Differences between PAR and VBW in the distances measured from OMI tip to the nearest root.

	Number of cases	Mean difference (mm)	Minimum difference (mm)	Maximum difference (mm)
PAR = VBW*	7	0.20	0.05	0.46
PAR > VBW	9	1.27	0.54	2.54
VBW > PAR	10	1.43	0.69	2.47
Total	26	1.05	0.05	2.54

* Differences smaller than 0.50 mm.

## DISCUSSION

The present study assessed how much the judgment of an observer, initially based on PAR only, would change when different radiographic images (i.e. VBW and/or OC) were additionally analyzed. The study did not aim to determine accuracy of radiographic methods, since in that case a CBCT image would probably have been needed to define it. Despite current concepts that a significant radiation dose reduction can be achieved for CBCT exams (i.e. by reducing, in general, image size and quality),^25^ doses are still higher when compared with intraoral radiographs, even when lower resolution and smaller fields of view are used.^26^ ^,^ ^27^ Following the ALARA (as low as reasonably achievable) principle and previous studies,^28^ ^,^ ^29^ the present study did not include CBCT images. Therefore, one limitation in the present results is that they do not point out the most accurate technique, but rather if there are differences among PAR only and PAR with additional radiographs (VBW and/or OR).

Subjective analysis showed that the addition of new radiographic techniques could alter observer judgement in many cases. They had different opinions when other radiographic techniques were added. PAR+VBW showed low to substantial agreement among observers, however the addition of OC seems to increase divergence between them. Both the opinion regarding OMI favorable position and the level of confidence of observers are impacted by the addition of different radiographs. However, it was not possible to determine a clear pattern of influence. Despite the absence of a definite pattern, with the addition of VBW, three observers changed their opinion regarding the OMI position whilst significantly increasing their confidence level (for two of them). On the other hand, when OC radiography was added, three observers also changed their judgment but the confidence levels of their responses decreased significantly for two of them. Confidence levels were more variable for ALL group, showing that more images are not necessarily better for evaluation. Overall, there is no consensus among observers on how much an additional radiograph impacts the confidence level.

Observers emitted their judgment about position of the OMI using a four-point scale without follow any criteria for this qualification. It means that the evaluation of each one of them was based on their subjective opinion and they followed their proper criteria. This was not considered a limitation, since the objective of this analysis was to determine if observers’ judgment could change when other images were added. This probably explains some very low Kappa values in the interobserver agreement analysis.

VBW and OC were always presented in conjunction with PAR, since the aim was to evaluate if other radiographic techniques could influence observer’s judgement. This may have inclined observers to actually maintain their opinion based on PAR. Despite this limitation, observers’ judgment changed because different perspectives were offer with extra images. Bitewing technique, due to its more orthogonal x-ray path, may avoid inadequate structure projections,^24^ favoring more straightforward image interpretations that changed observer judgement and influenced their confidence level. OC shows a different view of the OMI position,^14^ however due to image’s superposition (teeth crowns and/or fixed orthodontic devices), confidence levels did not increase in a significant number of cases when these radiographs were added.

Objective evaluation demonstrated that there were differences between measurements obtained on PAR and VBW in regard to the distance between OMI and the root (ICC= 0.22). Distances on PAR were greater than VBW in 9 cases, whilst distances were greater on VBW in 10 cases. In the remainder cases, distances were virtually the same. Even though it is not possible to conclude which of them would present the highest accuracy, and also considering the limitations of 2D image overlapping and distortion, results suggest that there is no advantage of one technique over the other, at least in a mesiodistal evaluation of the OMI position. Differently, Matzenbacher et al^15^ demonstrated that PAR had a greater degree of distortion than bitewing radiography; however, this study compared vertical measurements for the location of the implantation site of OMI (place where the head of the device will be located). This study did not evaluate horizontal measurements, nor did the region related to the OMI tip. Results of both studies could suggest that bitewing radiography might have an advantage over PAR in pre-placement evaluation, whereas in post-placement evaluation there would be no difference between two methods.

Accuracy of PAR for determining the final position of OMI is lower when compared with CBCT.^17^ ^,^ ^19^ The present study did not aim to test accuracy, however, results showed great variability suggesting low reliability of these radiographic methods (PAR, VBW and OC) for this type of evaluation. In general, it was clear that additional radiographs might influence the interpretation of the OMI final position, however, a definite pattern showing which exam modality overestimates or underestimates distances was not observed. 

Future studies designed to overcome limitations are recommended. Small interadicular spaces may present higher risk of damaging adjacent roots when OMI are placed. Confirming the position of the devices by means of radiographs is a routine procedure, however, in many cases some degree of uncertainty may remain from a single radiographic incidence. This study demonstrates that adding other radiographs to the conventionally used PAR does not necessarily help on diagnosis and decision making in cases of OMI; on the contrary, it could even generate more uncertainty. These results seem to indicate that CBCT could be the most reliable examination to evaluate the position of OMI, when there is suspicion of potential injuries of adjacent structures. Therefore, further investigations involving clinical follow-up and/or CBCT images with new methodological designs would be necessary to determine accuracy, sensitivity and specificity of radiographic examinations. 

Patients reported greater comfort during VBW technique. Additionally, since VBW displays both upper and lower regions in one exam, it is associated with lower radiation doses. It can be indicated in cases where OMI are placed in the same side of the maxilla or mandible. Presence of fixed appliance on the palate is a physical barrier for proper positioning of the film/sensor, representing a limitation for VBW technique. The addition of other radiographic techniques provides the clinician with new perspectives or views of the OMI. Even though it was not demonstrated how such exams could assist in determining the position of the device, the confidence level in judging this specific feature may increase with the addition of new radiographs, particularly VBW. It is important to stress however, that clinical perception and operator experience have a determinant role in this judgment, since no two-dimensional image will be able to provide conclusive information about the position of OMI. We consider that taking a radiograph after placing OMI is of particular importance to solve doubts about damage of adjacent structures, pain referred by patients during the procedure and adequate position of the device. Further investigations are needed.

## CONCLUSION

The null hypothesis proposed was rejected. Considering the limitations of the study and despite the absence of a definite pattern of judgements and confidence levels, it is concluded that the addition of other radiographic techniques (VBW and/or OC) may change the observers’ judgment about OMI final position without necessarily increasing the degree of certainty of their responses.
